# Evaluation of liver fibrosis in chronic hepatitis B patients with 2D shear wave elastography with propagation map guidance: a single-centre study

**DOI:** 10.1186/s12880-022-00777-7

**Published:** 2022-03-18

**Authors:** Seyhmus Kavak, Safak Kaya, Ayhan Senol, Nilgun Sogutcu

**Affiliations:** 1 Department of Radiology, Gazi Yasargil Training and Research Hospital, University of Health Sciences, Diyarbakir, Turkey; 2 Department of Infectious Diseases and Clinical Microbiology, Gazi Yasargil Training and Research Hospital, University of Health Sciences, Diyarbakir, Turkey; 3 Department of Pathology, Gazi Yasargil Training and Research Hospital, University of Health Sciences, Diyarbakir, Turkey

**Keywords:** Shear wave elastography, Propagation map, Liver fibrosis, Chronic hepatitis B

## Abstract

**Background:**

The aims of this study were to evaluate liver fibrosis with two-dimensional (2D) shear wave elastography (SWE) in patients with chronic hepatitis B (CHB), to compare 2D-SWE with histopathology and to determine the change in liver stiffness values after antiviral therapy.

**Material and methods:**

A total of 253 patients with CHB were included in this prospective study. 2D-SWE with propagation map guidance to measure liver stiffness, fibrosis-4 index (FIB-4) and aspartate aminotransferase to platelet ratio index (APRI) scoring and additional liver biopsy were performed in patients with CHB. Liver stiffness was measured again at 24 and 48 weeks in all patients. The Spearman rank correlation test was used to analyse the correlation between variables, and receiver operating curve analysis was used to evaluate the diagnostic performance in terms of fibrosis.

**Results:**

Liver stiffness measurements made with 2D-SWE demonstrated a significant positive correlation with the fibrosis stage and FIB-4 score (*r*_*s*_ = 0.774 and 0.337, respectively, *p* < 0.001 for both). The area under the curve value for kPa for the prediction of significant fibrosis was 0.956 (95% CI_s_) (0.920–0.991), and the optimal cut-off value was 8.2 kPa (sensitivity: 92.7% and specificity: 78.9%); these values were 0.978 (95% CI_s_, 0.945–1.000) and 10.1 kPa (sensitivity: 92.9% and specificity: 96.4%) for the prediction of severe fibrosis. After antiviral treatment, a decrease in liver stiffness values measured by 2D-SWE was detected (mean kPa values at 0 and 48 weeks; 9.24 and 7.36, respectively, *p* < 0.001).

**Conclusion:**

In conclusion, the measurement of liver stiffness with 2D-SWE has high diagnostic performance in the determination of hepatic fibrosis and can be used to evaluate the response to treatment in patients receiving antiviral therapy.

## Background

Chronic hepatitis B (CHB), with its complications such as cirrhosis and hepatocellular carcinoma, increases both mortality and morbidity and causes a significant financial burden. Therefore, early diagnosis and treatment are important. The aim of treatment in HBV-infected patients is to prevent the development of liver cirrhosis and hepatocellular cancer by providing viral suppression. In these patients, the HBeAg status, serum HBV DNA level, serum ALT levels, and liver biopsy results are important in demonstrating liver damage and in making treatment decisions [1, 2]. According to the European Association for the Study of the Liver (EASL) 2017 guidelines, there is an indication for fibrosis-independent treatment in patients with HBV DNA ˃ 20 000 IU/ml and ALT ˃ 2X the upper limit of normal [3]. However, it is important to determine liver damage in patients who are HBeAg positive or in those with HBV DNA ˃2000 IU/ml. In these patients, if there is moderate/severe necroinflammation or fibrosis in the liver, treatment is indicated (EASL 2017) [3]. The determination of liver damage is also valuable in determining the prognosis and in diagnosing cirrhosis and hepatocellular carcinoma [4]. Although liver biopsy is accepted as the gold standard in determining fibrosis, it may pose a risk and result in complications because it is an invasive procedure [5]; intra-abdominal bleeding, pneumothorax, haemothorax and biliary peritonitis are some of these complications. The frequency and severity of these complications are determined by the experience of the health care professionals performing the procedure and the suitability of the biopsy needle used. In some patients, life-threatening clinical scenarios may be encountered [6, 7]. Furthermore, fibrosis affects the liver parenchyma heterogeneously, and taking a biopsy sample that represents only a part of the whole organ and evaluating it is questionable at best. Another problem is that it is not always possible to perform a rebiopsy when necessary since the method is invasive. For these reasons, efforts have been focused on developing liver imaging methods and identifying serum biomarkers as alternatives to biopsy. In the EASL 2017 guidelines, it was emphasized that noninvasive tests should be used instead of direct biopsy, especially in patients with HBV DNA ˃ 2000 IU/ml and normal ALT levels [3]. Noninvasive procedures such as two-dimensional share wave elastography (2D-SWE), transient elastography (TE) and the use of serum biomarkers [especially the FibroMeter™, FibroTest, Hepascore and aspartate aminotransferase (AST) to platelet ratio index (APRI)] have been developed for this purpose [8].

Many parameters, such as age; body mass index (BMI); and serum levels of AST, alanine aminotransferase (ALT), gamma-glutamyl transpeptidase, alkaline phosphatase, cholesterol, albumin, bilirubin, platelets, alpha-2-macroglobulin, hyaluronic acid, haptoglobulin and apolipoprotein A1, have been used alone or in combination with one another as direct or indirect serum biomarkers of fibrosis. Noninvasive fibrosis markers, such as the Fibrosis-4 Index (FIB-4) (including age, AST, ALT and thrombocyte levels) and APRI, are two of the scoring methods recommended for use in clinical practice for this purpose [9–11]. However, the diagnostic values of FIB-4 and APRI scoring are limited [12].

As an imaging alternative, elastography has been used to measure liver fibrosis noninvasively. The method with the highest number of studies on liver elasticity measurement is TE, and there are studies showing that TE is useful in eliminating cirrhosis in patients with CHB and excluding fibrosis in inactive HBsAg carriers [13–16]. However, TE has some limitations (such as nonapplicability in patients with ascites, cholestasis, obesity and pregnancy and the small parenchymal area evaluated), and it has been reported to give unreliable results in 15.8% of patients [17–20]. Thanks to the newly introduced XL probe, these problems seem to have been partially solved by measuring from deeper areas. In another method, two-dimensional shear wave elastography (2D-SWE), unlike the measurement made from a single point in liver biopsy, a large number of regions of interest (ROIs) are placed in the liver parenchyma, and measurements are made from multiple points; thus, a larger area of the liver can be evaluated [21–23]. However, there is no consensus on objective criteria for confirming the reliability of measurements made in 2D-SWE techniques [24, 25]. Although there are many reports about the use of TE in patients with chronic hepatitis B in the literature, there are fewer publications on the use of 2D-SWE.

The aims of this study were to evaluate liver fibrosis with the 2D-SWE technique in HBeAg-negative chronic infection and patients with HBeAg-negative or HBeAg-positive chronic hepatitis with the help of a propagation map, to compare this method with histopathological results in patients with HBeAg-negative or HBeAg-positive chronic hepatitis, to evaluate fibrosis with 2D-SWE in patients before and after treatment and to compare these evaluations with the APRI/FIB-4 scoring systems to determine the suitability of 2D-SWE for use instead of biopsy.

## Materials and methods

This study was carried out prospectively in the Radiology Clinic of our hospital between January 2019 and October 2020. Informed consent was obtained from all patients, and the study was approved by the University of Health Sciences, Gazi Yaşargil Training and Research Hospital Institutional Ethics Committee (25.01.2019/215).

### Patient selection

A total of 253 patients with HBeAg-negative chronic hepatitis and HBeAg-negative or HBeAg-positive chronic infection who presented to the Infectious Diseases outpatient clinic between January 2019 and October 2020 were included in the study.

Patients with HBeAg-negative chronic infection (Group 1, N = 129) had been HBsAg positive for more than 6 months, with hepatitis B viral (HBV) DNA levels < 2000 IU/ml and normal ALT levels. There was no indication for treatment in these patients.

Patients with HBeAg-negative or HBsAg-positive chronic hepatitis (N = 109) had been HBsAg positive for more than 6 months, had HBV DNA levels > 2000 IU/ml, and had normal or high ALT levels. Liver biopsy was performed in these patients.

Patients with HBeAg-positive chronic infection (N = 15) had been HBsAg positive for more than 6 months, had HBV DNA levels > 10^7^ IU/ml, and had normal ALT levels. Since these patients had a family history of liver cirrhosis or hepatocellular cancer, there was an indication for treatment. For us to be able to provide treatment, liver biopsy was performed in these patients because biopsy was required in these patients in our country, and they were included in Group 2 (N = 124).

All patients were ≥ 18 years of age. Exclusion criteria were patients < 18 and > 65 years of age; those who had undergone transplantation; those who had previously undergone hepatobiliary surgery; those who had previously received oral antiviral therapy for CHB; those coinfected with hepatitis C, D or human immunodeficiency virus (HIV); and those with mass lesions in the liver, another chronic liver disease, diabetes mellitus, hypercholesterolemia, obesity (BMI ≥ 35), grade 2 or higher hepatosteatosis, alcoholism (> 2 glasses of alcohol consumption per day) or long-term hepatotoxic drug use. In addition, patients with baseline ALT levels greater than 5 times the upper limit were not included.

Information such as age, sex, weight, height and the duration of hepatitis B virus infection was recorded. BMI was calculated as body weight (kg)/height (m^2^). Haemogram, biochemical, complete hepatitis serological, anti-HIV, HBV DNA level, alpha-fetoprotein level, coagulation parameter and hepatobiliary US analyses were performed on all patients at the time of the first admission. The serum levels of platelets, ALT, AST, HBV DNA, hepatitis B e (HBe) antigen and antibody to HBe; international normalized ratio; prothrombin time; and activated partial thromboplastin time were recorded. In addition to the abovementioned serum biomarkers, hepatobiliary US and simultaneous shear wave imaging (SWI) were performed on the patients in both groups at 0, 6 and 12 months. Liver biopsy was also performed at 0 months for the patients in Group 2. Tenofovir disoproxil fumarate (TDF) or entecavir (ETV) treatment was randomly started for those who were indicated for treatment according to the biopsy results.

### Measurement of liver stiffness in 2D-SWE with propagation map guidance

**Fig. 1:**
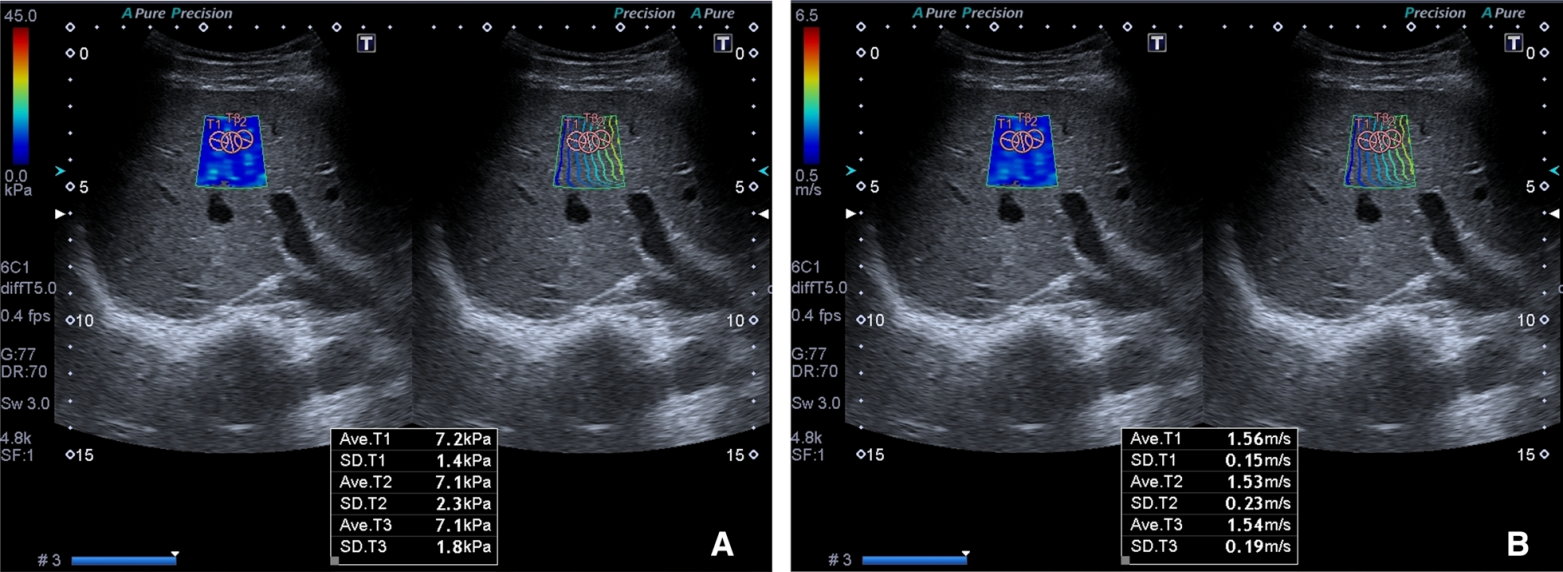
**A**, **B** Elastography and propagation map (**A**), shear wave speed map and propagation map (**B**) images of a 41-year-old woman with significant hepatic fibrosis

In all patients included in the study, an examination was performed by a radiologist (7 years of SWE experience) after at least 6 h of fasting to limit the effect of portal vein flow. 2D-SWE examinations were performed on traditional greyscale US evaluations and greyscale evaluations using a US system with a 1–6-MHz convex probe (Aplio 500, Canon Medical Systems, Tochigi, Japan) for diagnostic purposes. In the greyscale examination, the liver parenchymal echogenicity, parenchymal heterogeneity, contour irregularity, degree of liver fattening, presence of an incidental mass in the liver, portal vein diameter and thrombosis, long axis of the liver in the midclavicular line and spleen size were evaluated. Patients with incidental masses and portal vein thrombi were not included in the study. A ~ 2 × 3 cm sample box was placed on the greyscale image obtained from the right lobe of the liver with an intercostal approach while the patients were lying in the supine position and holding their breath for several seconds. At least three ROIs (1 cm^2^) were placed with their centre at least 2–5 cm below the Glisson capsule to prevent reverberation artefacts or subcapsular stiffness in line with the recommendations of the European Federation of Societies for Ultrasound in Medicine and Biology (EFSUMB) and the World Federation for Ultrasound in Medicine and Biology (WFUMB) [17–19, 25, 26]. At least 3 ROIs were placed in each sample box, and their average was taken. At least 5 sample boxes were created for each patient. The mean of the mean LSM values measured from all sample boxes was recorded. When we obtained a sample box full of data with the US system we used, a propagation map, an elastography map and a shear wave velocity map were automatically obtained simultaneously (Fig. 1). Measurements were obtained only from reliable ROIs (standard deviation values measured in elastography and shear wave velocity map < 30% of the measured mean value), in which the propagation map showed smooth and parallel lines.

### Liver biopsy and histopathology

Liver biopsy samples were obtained from the right lobe by an experienced radiologist under the guidance of a US device (Hitachi HI VISION Ascendus, Hitachi Medical Systems GmbH, Tokyo, Japan). Local anaesthesia was induced first, and subsequently, an 18-gauge Tru-cut needle was used. All liver biopsy samples were evaluated by the same pathologist, who had 15 years of experience. Liver biopsy samples with at least four portal areas were included in the study. The grading of necroinflammatory activity and the staging of fibrosis were performed using a histologically modified Ishak hepatitis activity index (HAI) scoring system. Later, Ishak scoring stages were transformed into METAVIR scoring stages. These patients were divided according to Ishak staging into five groups as follows: no fibrosis (Ishak F0 = METAVIR F0), mild fibrosis (Ishak F1, F2 = METAVIR F1), moderate fibrosis (Ishak F3 = METAVIR F2), severe fibrosis (Ishak F4, F5 = METAVIR F3) and cirrhosis (Ishak F6 = METAVIR F4) [27].

### Biochemical scoring

The demographic and biochemical data of the patients included in the study were age, platelet count and serum AST and ALT levels. The tests were performed in our hospital's biochemistry laboratory. The FIB-4 score was calculated using Sterling's formula as follows: age (year) × AST [U/L]/(platelet [10^9^/L] × (ALT [U/L])^1/2^) [28]. The APRI score was calculated using Wai's formula (AST/upper normal limit 40 IU/L)/thrombocyte count (thrombocyte × 10^9^/L) × 100 [29].

### Statistical analysis

All analyses were carried out using SPSS version 23 software. The Shapiro–Wilk test was applied to examine the distribution normality of continuous data. The Kruskal–Wallis and Mann–Whitney U tests were used for continuous variables, while the chi-square or Fisher’s exact test was used for categorical variables in comparisons between the different groups. The Wilcoxon signed rank test was used for the comparison of nonnormally distributed variables at the different time points in each group. Correlations between variables were assessed using the Spearman rank correlation test. The optimal cut-off values of the SWI (kPa and m/s), APRI score and FIB-4 score for the prediction of patients with different levels of disease severity (significant and severe fibrosis) were calculated by applying receiver operating curve (ROC) analysis. A value of *p* < 0.05 was considered statistically significant.

## Results

A total of 253 patients [139 (54.9%) male and 114 (45.1%) female patients], with a mean age of 34.6 (interquartile range 26–42), were included in the study. The age and sex comparisons of the groups are given in Table 1. In Group 2, the METAVIR scores of 10.5% of the patients were F0, 31.5% were F1, 37.1% were F2 and 21% were F3.

**Table 1:** Baseline characteristics of the groups (n = 253)

	Group 1 (n = 129)	Group 2 (n = 124)	*p* value
Age (years)	35.5 (26.0–44.0)	33.7 (25.0–41.0)	0.282
Gender (male)	69 (53.5)	70 (56.5)	0.746
Laboratory values			
HBV DNA (IU/ml)	405.5(35.0–567.0)	9.9 × 10^6^(5.8 × 10^3^–1.5 × 10^5^)	< 0.001
ALT	21 (17–28)	28 (19–41)	< 0.001
AST	20 (17–24)	28 (19–30)	< 0.001
Platelet (10^9^/L)	257 (215–296)	218 (196–246)	0.002
INR	1.12 (1.08–1.19)	1.10 (1.05–1.16)	0.155
PT (s)	12.0 (11.5–12.6)	11.8 (11.2–12.5)	0.581
PTT (s)	29.6 (28.4–31.4)	30.2 (28.6–32.1)	0.041
Biochemical scoring			
FIB-4	0.57 (0.43–0.75)	0.75 (0.44–0.96)	0.227
APRI	0.22 (0.17–0.28)	0.35 (0.20–0.47)	< 0.001
Liver diameter (mm)			
Right lobe	146.0 (140.0–153.0)	147.0 (140.0–155.0)	0.412
Left lobe	70.0 (64.0–79.0)	70.0 (65.0–77.0)	0.717
Spleen diameter (mm)	109.0 (101.0–118.0)	111.5 (102.0–120.0)	0.464
2D-SWE, LSM			
kPa	6.2 (5.6–6.7)	8.15 (6.8–9.4)	< 0.001
m/s	1.41 (1.34–1.49)	1.59 (1.48–1.83)	< 0.001

Quantitative data were expressed as median (25–75% quartiles) and qualitative data were expressed as number and percentage (%)

*Group1* HBeAg negative chronic infection carriers, *Group 2* HBeAg negative or positive chronic hepatitis and HBeAg positive chronic inection patients, *ALT* Alanine aminotransferase, *AST* Aspartate aminotransferase, *INR* International normalized ratio, *PT* Prothrombin time, *PTT* Partial thromboplastin time, *FIB-4* Fibrosis 4, *APRI* AST-to platelet ratio index, *2D-SWE* Two dimensional share wave elastography, *LSM* Liver stiffness measurement

Baseline FIB-4 scores were similar between the two groups (*p* = 0.227), whereas APRI scores and liver stiffness measurement (LSM) values (2D-SWE measurements in kPa and m/s) were significantly higher in Group 2 than in Group 1 (*p* < 0.001 and *p* < 0.001, respectively). The results of the correlation analyses are shown in Table 2. Liver stiffness measured by 2D-SWE (kPa, m/s) showed a significant positive correlation with the HAI score, fibrosis stage and FIB-4 score. However, there was no significant correlation between the APRI score and 2D-SWE measurements.

**Table 2:** Correlation between liver stiffness measurements and serum fibrosis markers at baseline

	LSM (kPa)		LSM (m/s)	
	*r*_*spearman*_	*p* value	*r*_*spearman*_	*p* value
HAI score	0.528*	0.001	0.523*	0.001
Fibrosis stage	0.788**	< 0.001	0.776**	< 0.001
FIB-4 score	0.339*	0.001	0.335*	0.001
ALT (U/L)	0.062	0.491	0.075	0.409
AST (U/L)	0.158	0.079	0.153	0.091
Platelet (10^9^/L)	− 0.069	0.448	− 0.037	0.457
APRI score	0.149	0.099	0.134	0.139

Data are presented as r_s_ (p)

r_s_ means Spearman’s ranks correlation coefficient (*Moderate correlation, **Very strong correlation), *LSM* Liver stiffness measurement, *HAI* Hepatitis activity index, *FIB-4* Fibrosis 4, *ALT* Alanine aminotransferase, *AST* Aspartate aminotransferase, *APRI* AST-to platelet ratio index

Comparisons between the rates of change in LSM values between patients with baseline LSM ≥ 8.2 kPa and those with baseline LSM < 8.2 kPa, according to antiviral treatment duration and treatment regimen, are shown in Tables 3 and 4. The relative (%) LSM reduction was significantly higher in the LSM ≥ 8.2 kPa group than in the LSM < 8.2 kPa group at 24 and 48 weeks compared to baseline (*p* < 0.001 and *p* < 0.001, respectively).

**Table 3:** Change in serum biomarkers and liver stiffness over time during antiviral therapy*

	Baseline	24.week	48.week
LSM, 2D-SWE (kPa)	9.24 (5.8–19.9)	8.01 (6.1–17.5)^a^	7.36 (5.4–15.0)^a,b^
LSM, 2D-SWE (m/s)	1.79 (1.34–2.65)	1.57 (1.34–2.43)^a^	1.44 (1.33–2.21)^a,b^
APRI	0.39 (0.12–1.93)	0.32 (0.11–1.44)^a^	0.30 (0.06–1.15)^a^
FIB-4	0.83 (0.17–3.22)	0.79 (0.16–3.64)^a^	0.75 (0.16–3.96)^a^
ALT (U/L)	45.1 (5–195)	32.1 (8–168)^a^	30.5 (7–144)^a^
AST (U/L)	30.9 (13–157)	25.7 (12–98)^a^	25.6 (6–72)^a^
INR	1.12 (0.96–1.38)	1.13 (0.97–1.77)	1.13 (0.99–1.36)
PLT (10^9^/L)	201 (69–364)	212 (74–427)	223 (94–373)
HBV DNA (IU/ml)	95,816,415	127732^a^	794^a^^,b^

*LSM* Liver stiffness measurement, *2D-SWE* Two dimensional share wave elastography, *APRI* AST-to platelet ratio index, *FIB-4* F ibrosis 4, *ALT* Alanine aminotransferase, *AST* Aspartate aminotransferase, *INR* International normalized ratio, *PLT* Platelet

^a^*p* < 0.005 compared to baseline

^b^*p* < 0.005 compared to week 24

*It included 84 patients under antiviral therapy

**Table 4:** Liver stiffness measurement changing according to duration of antiviral therapy

	Baseline vs week24				Baseline vs week48			
	Overall n = 84	LSM $\ge$ 8.2 n = 61	LSM < 8.2 n = 23	*p* value	Overall n = 84	LSM $\ge$ 8.2 n = 61	LSM < 8.2 n = 23	*p* value
LSM decrease (%)	14.5 (3.1–22.8)	15.2 (6.0–22.8)	12.6 (3.1–20.9)	< 0.001	22.1 (2.1–37.8)	23.2 (5.2–37.8)	19.2 (2.1–33.4)	< 0.001
*Overall LSM decrease*								
> 10%, n (%)	47 (55.9)	37 (60.6)	10 (43.5)	0.008	50 (59.5)	37 (60.6)	13 (56.5)	0.811
> 20%, n (%)	9 (10.7)	7 (11.5)	2 (8.6)	0.024	22 (26.2)	18 (29.5)	4 (17.4)	< 0.001

*LSM* liver stiffness measurement, *kPA* kilopascal

**Fig. 2:**
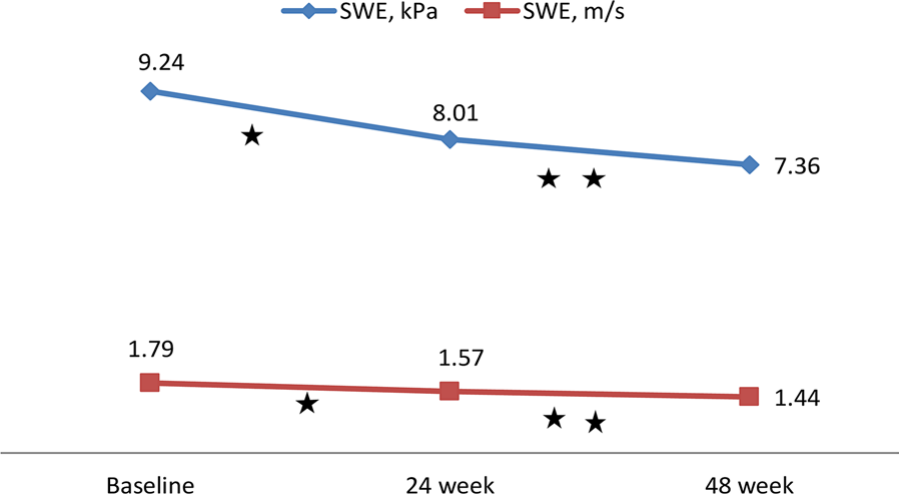
Comparison of liver stifness measurements by SWE (kPa and m / s) during antiviral therapy. *There is a statistically significant decrease compared to the baseline value at the 24th week (*p* < 0.05). **There was a statistically significant decrease in the 48th week compared to the 24th week and the baseline value (*p* < 0.05)

**Fig. 3:**
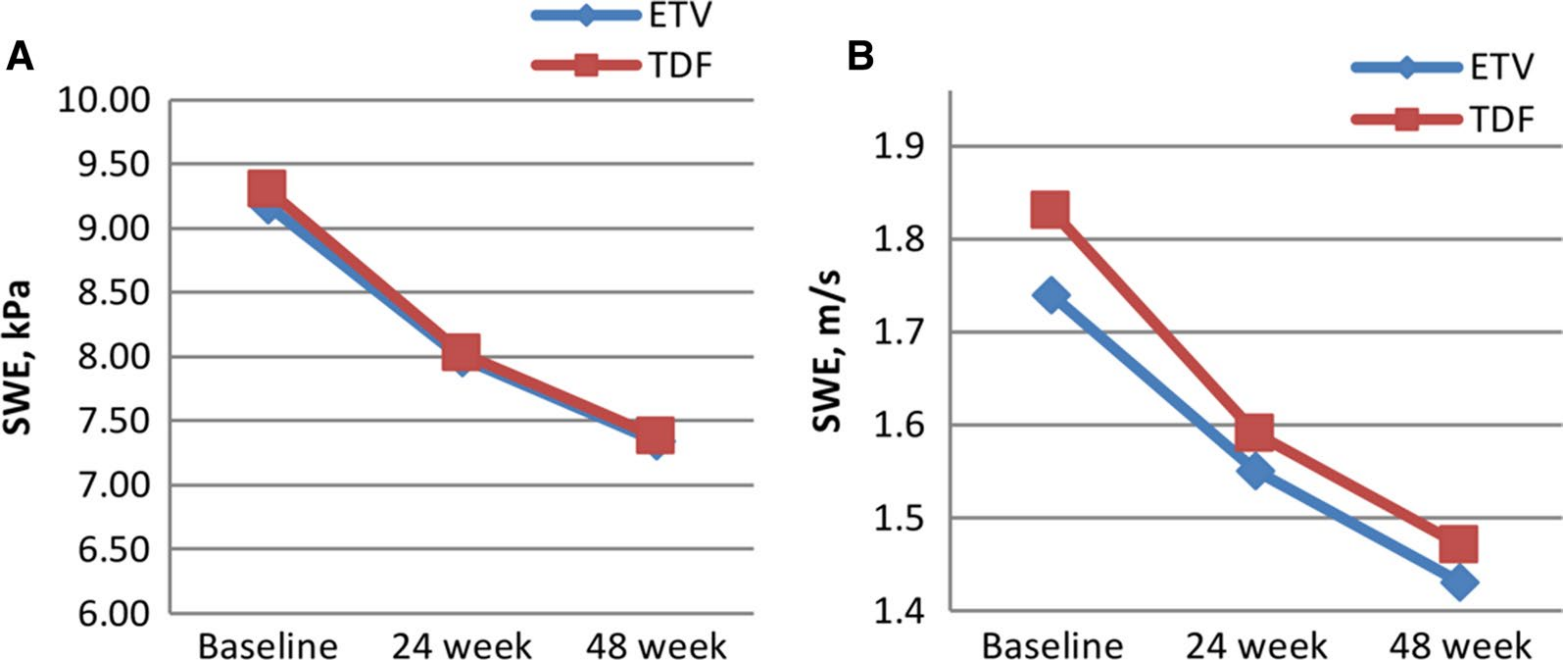
**A, B**: Change of liver stiffness measurement (SWE: kPa (**A**) and m/s (**B**)) levels over time according to different treatment regimens

**Fig. 4:**
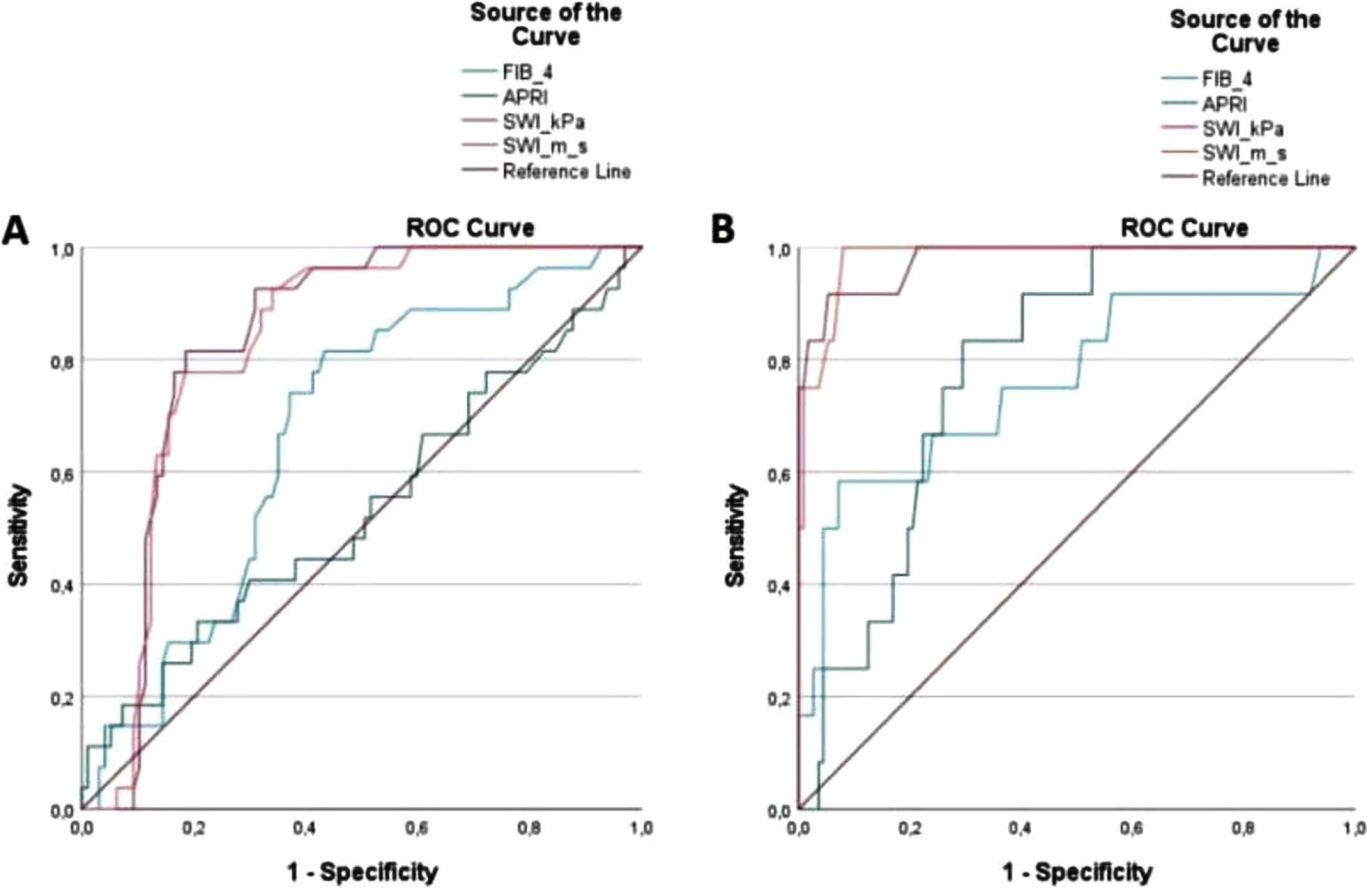
ROC curves for APRI, FIB-4 and shear wave imaging (SWI: kPa and m/s) for predicting **A** significant fibrosis (F2), **B** severe fibrosis (F3)

In Group 2, there was a significant decrease in kPa and m/s values measured with 2D-SWE at 24 and 48 weeks compared to the initial measurements (Fig. 2). Similar rates of decline were observed in kPa and m/s values measured by 2D-SWE with the two different treatment regimens (Table 5, Fig. 3). For predicting patients with F2 scores, the area under the curve value for 2D-SWE measurements for kPa was 0.956 [95% confidence interval (CI) 0.920–0.991] and that for m/s was 0.952 (95% CI 0.913–0.990). These values were 0.978 (95% CI 0.945–1.000) for kPa and 0.982 (95% CI 0.961–1.000) for m/s for predicting patients with F3 (Fig. 4). The optimal cut-off value measured by 2D-SWE was 8.2 kPa (sensitivity: 92.7% and specificity: 78.9%) for predicting patients with F2 and 10.1 kPa (sensitivity: 92.9% and specificity: 96.4%) for predicting patients with F3. The optimal cut-off values of kPa and m/s measured by 2D-SWE for predicting patients with F2 and F3 are shown in Table 6.

**Table 5:** Liver stiffness measurement changing according to treatment regimen

	Baseline versus week24				Baseline versus week48			
	Overall (n = 84)	ETV (n = 44)	TDF (n = 40)	*p* value	Overall (n = 84)	ETV (n = 44)	TDF (n = 40)	*p* value
*LSM decrease*								
kPa value*	1.23 (0.3–2.8)	1.19 (0.2–2.4)	1.27 (0.2–2.8)	0.098	1.88 (0.1–4.8)	1.84 (0.1–3.5)	1.92 (0.1–4.8)	0.174
kPa %*	13.3 (3.1–22.8)	13.1 (2.1–22.8)	13.4 (2.3–21.7)		20.3 (2.1–37.8)	19.9 (2.7–36.5)	20.8 (2.1–37.8)	
m/s value*	0.22 (0.03–0.31)	0.21 (0.02–0.30)	0.23 (0.02–0.31)	0.734	0.35 (0.03–0.44)	0.33 (0.03–0.39)	0.37 (0.04–0.44)	0.234
m/s %*	12.3 (1.9–21.9)	11.7 (1.9–20.6)	12.9 (2.6–21.9)		19.6 (1.4–38.6)	18.4 (1.4–35.3)	20.7 (1.5–38.6)	
*LSM decrease*								
> 10%, n (%)	47 (55.9)	25 (56.8)	22 (55.0)	0.802	50 (59.5)	27 (61.4)	23 (57.5)	0.588
> 20%, n (%)	9 (10.7)	5 (11.4)	4 (10.0)	0.659	22 (26.2)	12 (27.3)	10 (25.0)	0.915

*ETV* Entecavir, *TDF* Tenofovir disoproxil fumarate, *LSM* Liver stiffness measurement, *kPa* Kilopascal, *m/s* Meter/second (*The mean kPa and m/s values measured with two dimensional share wave elastography and the minimum and maximum values are given)

**Table 6:** Comparison of area under curve (AUC) to predict the disease severity

	AUC (95%Cl)	Cut-off levels	Sensitivity	Specificity	PPV	NPV	*p* value
F2							
2D-SWE, kPa	0.956 (0.920–0.991)	8.2	0.926	0.788	0.786	0.927	< 0.001
2D-SWE, m/s	0.952(0.913–0.990)	1.65	0.926	0.741	0.775	0.905	< 0.001
APRI	0.657(0.549–0.765)	0.56	0.667	0.392	0.571	0.701	0.063
FIB-4	0.743(0.648–0.838)	0.82	0.741	0.588	0.471	0.710	< 0.001
F3							
2D-SWE, kPa	0.978(0.945–1.000)	10.1	0.929	0.964	0.812	0.991	< 0.001
2D-SWE, m/s	0.982(0.961–1.000)	1.91	0.929	0.945	0.722	0.991	< 0.001
APRI	0.787(0.688–0.886)	0.86	0.883	0.705	0.375	0.905	0.001
FIB-4	0.765(0.601–0.929)	1.03	0.750	0.616	0.368	0.933	0.001

*PPV* Positive predictive value, *NPV* Negative predictive value, *F2* Predict significant fibrosis, *F3* Predict severe fibrosis, *2D-SWE* Two dimensional shear wave elastography, *kPa* kilopascal, *m/s* meter/second, *APRI* Aspartate aminotransferase to platelet ratio index, *FIB-4* Fibrosis-4

## Discussion

In this study, we aimed to estimate the degree of liver fibrosis using a 2D-SWE method in patients infected with HBV and to reveal the change in liver stiffness measurements made with 2D-SWE after antiviral drug therapy. Looking at the overall results, we found that LSMs made with 2D-SWE could predict liver fibrosis at very high sensitivity and specificity for METAVIR scores F2 and F3 when the gold-standard liver biopsy was considered. In addition, we determined that at 24 and 48 weeks after antiviral treatment, the LSM values ​obtained with 2D-SWE decreased statistically significantly compared to the pretreatment values.

In the present study, when Group 1 and Group 2 were compared, it was observed that there was a significant increase in APRI scores in Group 2 compared to Group 1 (*p* < 0.001). In addition, the kPa value measured by 2D-SWE was significantly higher in Group 2 than in Group 1. The measured kPa and m/s values showed a significant positive correlation with the HAI score, fibrosis stage and FIB-4 score. The results obtained by 2D-SWE yielded a cut-off value of 8.2 kPa for F2 staging with a sensitivity of > 92% and a cut-off value of 10.1 kPa for F3 staging with a sensitivity of 92.9%. Bende et al. investigated the success of 2D-SWE in predicting liver fibrosis using TE as a control method in their study of 171 patients with or without chronic hepatopathy. In this study, the best 2D-SWE threshold value was found to be 6.9 kPa (sensitivity, 85.8%; specificity, 90.2%) for F ≥ 2 and 8.2 kPa (sensitivity, 87.5%; specificity, 86.8%) for F ≥ 3 [30]. In a study by Leung et al., it was reported that F2 could be defined by 2D-SWE using a cut-off value of 7.1 kPa with 92.1% specificity and 84.7% sensitivity in patients with CHB [31]. In a study by Lee et al., good results were obtained for the definition of hepatic fibrosis and cirrhosis; however, it was stated that this study had limitations such as a heterogeneous population and liver biopsy not having been performed [32]. Jeon et al. performed 2D-SWE measurements with the guidance of a propagation map and reported a cut-off value of 8.1 kPa for F2 with 94.1% sensitivity and 95.8% specificity [33]. In studies where METAVIR-based fibrosis scoring was used as the gold standard, the cut-off values for different stages of fibrosis calculated for 2D-SWE measurements made in kPa and m/s vary according to the 2D-SWE technique used. Even in studies performed with the same measurement device model, although a certain level of measurement congruence is observed in correlation studies between different operators, the cut-off values obtained for kPa and m/s may differ. Hence, we believe that the use and development of applications such as the propagation map provided by the system used in the present study might be important to ensure measurement congruence between operators using the same device.

As a result of biopsy performed in the group of chronic hepatitis B patients (N = 124), 84 patients were eligible for antiviral therapy, and oral antiviral (ETV or TDF) treatment was initiated. The mean LSM values in kPa and m/s measured by 2D-SWE at 24 and 48 weeks in these patients were significantly lower than the baseline mean LSM value. In patients who started oral antiviral therapy, the mean kPa value measured before treatment was 9.24; it was 8.01 at the 24th week and 7.36 at the 48th week. We found that the measured kPa values at the 48th week decreased more than 10% in 50 patients (59.5%) and more than 20% in 22 patients (26.2%). There are studies investigating the dynamic change in LSM values, which represent liver stiffness and partial liver fibrosis, with antiviral therapy, usually using TE, in chronic hepatitis B patients [34, 35]. Liang et al. measured the degree of fibrosis with liver biopsy at baseline and at the 24th week, 52nd week and 104th week and LSM values with TE in 164 chronic hepatitis B patients who started ETV treatment. They found that the average LSM value at the 24th week showed a rapid decrease from 8.6 to 6.1 kPa, accompanied by a decrease in ALT, and in later measurements, the rate of decrease in the average LSM value slowed down significantly until the 104th week [34]. In their study, in which Wu et al. evaluated 120 chronic hepatitis B patients and used TE, they measured the initial mean LSM value as 13.8 kPa before antiviral treatment (ETV) and a value of 10.4 kPa at the 26th week. They reported that the decrease in the average LSM value continued at the 52nd and 78th weeks. In the same study, according to 78th-week biopsy samples, 54 (45%) patients had a regression in the degree of fibrosis with ETV treatment, and the authors suggested that a 40% reduction in LSM values was an important determinant of fibrous regression (AUC = 0.69, sensitivity 69% and specificity 68%) [35]. Similar to our study, the decrease in the average LSM values in both studies was more pronounced in the first 6 months. The first reason for this can be explained by the high degree of pretreatment necroinflammation. The difference in the mean LSM decline between studies may be due to the proportional difference in fibrosis grades among the patients studied. Although the clinical outcomes of CHB patients improve with effective antiviral therapy, antiviral therapy cannot completely eliminate the risk of developing HCC [36]. The underlying fibrotic burden is independently predictive of HCC development [37]. Therefore, hepatic fibrosis staging or the measurement of liver stiffness should be dynamically evaluated for effective surveillance of CHB patients receiving antiviral therapy. The large Korean cohort study of 1130 chronic hepatitis B (CHB) patients confirmed the longitudinal role of TE in evaluating the development of HCC. In this study, TE values as an independent risk factor were defined as a 3.07-fold increased risk of developing HCC at 8–13 kPa and 6.60-fold at > 23 kPa compared with an LSM value of < 8 kPa as a reference [38]. Kim et al. investigated the prognostic role of TE in predicting liver-related events (LREs), such as HCC, hepatic decompensation, and the development of cirrhosis, in their study with 128 patients with CHB. They found that the probability of developing LREs in patients with TE values > 19 kPa was significantly higher than that in patients with TE values ≤ 19 kPa (HR, 7.176; *p* = 0.001) [39].

The absence of liver biopsy results after treatment is a limitation of this study. In future studies, comparing the results of posttreatment biopsy with the measurements obtained by the 2D-SWE method in patients receiving treatment will provide results that are more meaningful. Moreover, we found that the APRI and FIB-4 scoring systems were weak in determining fibrosis and did not show a positive correlation with LSMs.

In conclusion, LSMs made with 2D-SWE with propagation map guidance provide high diagnostic performance in the assessment of liver stiffness. The use of 2D-SWE with propagation map guidance could prevent the performance of unnecessary biopsies by identifying patients who require biopsy. In addition, 2D-SWE can guide patient management by providing both the follow-up of chronic hepatitis B patients and the evaluation of their response to antiviral treatment. Similar to US analysis, 2D-SWE could become a part of routine follow-up in the near future.

## Data Availability

The datasets used and/or analysed during the current study available from the corresponding author on reasonable request.

## Abbreviations

APRI: Aspartate aminotransferase to platelet ratio index
BMI: Body mass index
EASL: The European Association for the Study of the Liver
EFSUMB: European federation of societies for ultrasound in medicine and biology
ETV: Entecavir
HAI: Hepatitis activity index
SWI: Shear wave imaging
TDF: Tenofovir disoproxil fumarate
TE: Transient elastography
WFUMB: World federation for ultrasound in medicine and biology
2D-SWE: Two-dimensional shear wave elastography
